# Diabetic Ketoacidosis Presenting with Pseudonormoglycemia in a 15-Year-Old Girl with Type 1 Diabetes Mellitus

**DOI:** 10.4274/Jcrpe.905

**Published:** 2013-05-30

**Authors:** Sinem Akbay, Arda Yel, Ülkü Yıldırımer, Şule Can, Bumin Dündar

**Affiliations:** 1 İzmir Tepecik Training and Research Hospital, Department of Pediatric Endocrinology, İzmir, Turkey; 2 İzmir Katip Çelebi University Faculty of Medicine, Department of Pediatric Endocrinology, İzmir, Turkey

**Keywords:** diabetic ketoacidosis, hyperlipidemia, pseudonormoglycemia

## Abstract

Pseudonormoglycemic diabetic ketoacidosis (DKA) is a rare condition and has been reported only in a few adult patients. We present a 15-year-old girl with a 9-year history of type 1 diabetes who presented with euglycemic and extreme hypertriglyceridemia. The acidosis and hypertriglyceridemia resolved with intravenous insulin therapy and rehydration. Hyperlipidemia was the apparent cause of pseudonormoglycemia in this patient. The findings in the present case demonstrate that also in children, DKA can rarely occur without abnormal blood glucose levels. Assessment of the acid-base status, urinary glucose, and ketone readings is therefore important in all diabetic patients who are unwell at admission and have normal glucose levels. In such patients, hyperlipidemia may cause pseudonormoglycemia. An awareness of this rare treatable life-threatening condition is important.

**Conflict of interest:**None declared.

## INTRODUCTION

Diabetic ketoacidosis (DKA) is a state of absolute or relative insulin deficiency (1,2,3,4). Hyperglycemia, acidosis, ketonemia, and ketonuria constitute the biochemical criteria for diagnosis of DKA (4). Despite elevated blood glucose levels, accepted as necessary in the definition of DKA, unusual cases with only modestly increased blood glucose concentrations have been reported, and the term euglycemic ketoacidosis is used to define these cases (2,3,4). Pseudonormoglycemic DKA is another term used for the few adult patients with type 1 diabetes mellitus (T1DM) reported to present with hyperlipidemia and normoglycemic blood levels (1,5,6,7).

We present a 15-year-old girl with pseudonormoglycemic DKA due to hyperlipidemia. To our knowledge, there are no previous reports of pediatric cases with pseudonormoglycemic DKA.

## CASE REPORT

A 15-year-old girl with a 9-year history of T1DM with poor control and an 8-year history of celiac disease was admitted to the pediatric emergency department with complaints of nausea, dizziness, and vomiting. She was on a basal bolus insulin regimen, but it was reported that the treatment was not regularly administered. The patient’s prenatal/natal/postnatal history was unremarkable. Her five siblings were healthy. She had a history of many prior hospitalization episodes because of diabetes and celiac disease.On initial assessment, the patient appeared to be unwell, exhausted, and had Kussmaul breathing. Her weight was calculated as -3.56 standard deviation score (SDS) and her height as -2.03 SDS. Her body mass index SDS was 1.01. She was clinically moderately dehydrated with dry buccal mucosa, reduced skin turgor, and depressed emotional state. Her cardiovascular, respiratory, abdominal, and neurological examinations were normal, and no obvious sign of infection was identified. DKA was suspected and presence of metabolic acidosis was confirmed with a pH of 7.08, bicarbonate concentration of 4.4 mEq/L, pCO2 of 15 mmHg, and an elevated anion gap of 37.3 mEq/L. Urinalysis obtained from a second-voided urine sample showed 3+ ketonuria and 4+ glycosuria; these findings were confirmed by a repeat test within the same hour. On the other hand, the patient’s venous blood glucose concentration was 115 mg/dL (6.38 mmol/L), and her capillary blood glucose obtained by stick was 122 mg/dL (6.77 mmol/L). Hemoglobin A1c was 9% (normal range: 4-6%).

The patient was diagnosed as a case of euglycemic DKA. Following loading with 20 mL/kg 0.9% saline for rehydration in the emergency department, the blood glucose level by stick was 35 mg/dL. Following administration of 2 mL/kg of 10% dextrose intravenously, the blood glucose level rose to 45 mg/dL. The patient was transferred to the pediatric service for advanced clinical care. At this time, she was evaluated to be moderately dehydrated, and intravenous fluid therapy (3000 mL/m2/day) was started. The dextrose content of this fluid was adjusted to 10% to maintain euglycemia. When the blood glucose reached 77 mg/dL, intravenous insulin infusion (0.1 IU/kg per hour) was started to resolve the acidosis. Blood glucose levels were monitored hourly and detected to be between 55 and 78 mg/dL. The acidosis gradually improved, and her blood gas levels became normal at the fourth hour of the treatment, at which time the IV insulin and dextrose-saline infusion were stopped. Subcutaneous insulin regimen was started, and the patient began to take oral fluids. There was no history to explain the state of euglycemia in this diabetic patient. However, the analysis of her blood sample taken at admission revealed a total cholesterol level of 232 mg/dL, (6 mmol/L) (N=110-199 mg/dL), triglyceride level of 427 mg/dL (4.82 mmol/L) (N=30-199 mg/dL), and a HDL cholesterol level of 56 mg/dL (1.45 mmol/L) (N=40-85 mg/dL). LDL cholesterol level could not be calculated because of a triglyceride level >400 mg/dL. Hyperlipidemia was speculated to be the cause of the spurious normoglycemia noted in this patient. Through educational sessions held with the patient and her family members, they were instructed to improve the nutritional intake and the insulin regimen of the patient. The patient’s lipid profile was found to have reverted to normal at the follow-up visit after discharge. 

## DISCUSSION

DKA is a state of absolute or relative insulin deficiency (2,3,4). In the healthy person, there is a balance between the insulin levels and the stress hormones which include glucagon, cortisol, epinephrine, and growth hormone. When insulin is absent or its level is low, this homeostasis is lost. This results in a state of excess of the stress hormones causing hyperglycemia, osmotic diuresis, ketosis, and acidosis. Insulin inhibits glycogenolysis and gluconeogenesis. Deficiency of insulin and excess of stress hormones lead to mobilisation of free fatty acids from adipose tissue with resultant ketogenesis and ketoacidosis (3,8,9).

The biochemical criteria for diagnosis of DKA are hyperglycemia (blood glucose level >11 mmol/L), acidosis (venous pH value <7.3 and serum bicarbonate level <15 mmol/L), ketonemia, and ketonuria (4). Although DKA is generally associated with significantly elevated blood glucose levels, only modestly increased blood glucose concentrations can also be encountered, and this state is defined as euglycemic ketoacidosis (2,3,4). This type of DKA was first described in adults by Munro et al (10) in 1973, after analysis of 211 episodes of DKA. Of these episodes, 37 were described as euglycemic (defined as a blood glucose level of 300 mg/dL (16.7 mmol/L) or lower and a plasma bicarbonate level of 10 mmol/L or lower at presentation). In 1993, Jenkins et al (11) reported a series of 722 episodes of DKA, of which 23 episodes (3.2%) were described as euglycemic, based on the same diagnostic criteria as above. These authors reported that true euglycemic ketoacidosis was rare, occurring in 0.8-1.1% of all episodes. It has been suggested that glucose readings of 200 mg/dL (11 mmol/L) be used as a cut-off value for defining true euglycemic DKA in adults (2,12).

Although euglycemic DKA is rare, low caloric intake, starvation, persistent vomiting, and pregnancy can be contributory factors in its development (2,5,9,13). Fasting frequently occurs during the development of DKA either as a result of an underlying condition such as infection or as a result of the worsening of ketoacidosis per se. Fasting leads to reduced carbohydrate intake, to decreased glucose production, or to increased glucose utilization. It predisposes patients with T1DM to euglycemic ketoacidosis (9,14). During pregnancy, euglycemic DKA may develop more rapidly than it does in non-pregnant individuals since pregnancy predisposes the mother to accelerated starvation with enhanced lipolysis, which can result in ketonuria after an overnight fast. Thus, this state is referred to as the “euglycemic DKA” of pregnancy. These women also have a lower buffering capacity due to the progesterone-induced respiratory alkalosis resulting in a compensatory metabolic acidosis. Furthermore, euglycemic DKA is not uncommon in pregnancy due to a tendency for ketosis and also due to glomerular hyperfiltration which causes glycosuria at lower serum glucose levels (14). Glycogen storage disorders, chronic liver disease resulting in decreased glycogen stores can also cause euglycemic ketoacidosis and must be considered in the differential diagnosis (5,9).

It is well known that blood glucose level is not an accurate indicator of severity of DKA, since DKA can present with normal plasma glucose concentrations; however, DKA presenting with true euglycemia is not a common occurrence (5,8). When a patient presents with normoglycemia and DKA, pseudonormoglycemia should also be ruled out. Rumbak et al (1) reported pseudonormoglycemia in a 24-year-old case with DKA, and they suggested that the urine glucose rather than blood glucose might be a better index of response to intravenous insulin and should therefore be monitored more closely than usual. Indeed, our patient had 4+ glycosuria despite normal serum glucose levels, and we thought that she might have pseudoeuglycemia. Jenkins et al (11) emphasized the importance of ketone testing rather than glucose testing in the diagnosis of ketoacidosis. It is also known that extreme hyperlipidemia can result in lipemic serum with a spurious lowering of blood glucose (pseudonormoglycemia) and of blood sodium (pseudohyponatremia) (1,5,6,7). High levels of circulating lipids, because of volume displacement, may lead to a decrease in the plasma concentration of glucose, thus to a delay in the diagnosis of DKA (1). Also, in insulin deficiency, hepatic production of apoprotein B-containing lipoproteins increases and triglycerides cannot be adequately cleared from the plasma due to decreased activity of insulin-dependent lipoprotein lipase (LPL), and this results in hypertriglyceridemia. In insulin-dependent diabetes, effective treatment of hyperglycemia usually normalizes serum lipids (1,15,16).

In our patient, there was no history of prolonged starvation, possibility of pregnancy, or chronic liver disease to explain the euglycemia accompanying the metabolic acidosis, glycosuria and ketonuria. Also, high concentrations of triglyceride, cholesterol, LDL, and VLDL were detected, probably due to inadequate insulin treatment, poor control of diabetes, and unhealthy nutritional intake. We thought the hyperlipidemia could be a cause of the factitious normoglycemia. Indeed, with treatment, glycosuria and ketonuria returned to normal. Also, the hyperlipidemia also had resolved at the follow-up visit.

In conclusion, the findings in the present case demonstrate that also in children, DKA can rarely occur without abnormal blood glucose levels. Therefore, assessment of the acid-base status, urinary glucose, and ketone readings is important in all diabetic patients who are unwell at admission and have normal glucose levels. In such cases, hyperlipidemia may cause pseudonormoglycemia. An awareness of this life-threatening but treatable condition is important. 
